# Magnetofluoro-Immunosensing Platform Based on Binary Nanoparticle-Decorated Graphene for Detection of Cancer Cell-Derived Exosomes

**DOI:** 10.3390/ijms23179619

**Published:** 2022-08-25

**Authors:** Jaewook Lee, Ji-Heon Lee, Jagannath Mondal, Joon Hwang, Han Sang Kim, Vinoth Kumar, Akhil Raj, Seung Rim Hwang, Yong-Kyu Lee

**Affiliations:** 1 4D Convergence Technology Institute (National Key Technology Institute in University), Korea National University of Transportation, Jungpyeong 27909, Korea; 2 Department of Chemical and Biological Engineering, Korea National University of Transportation, Chungju 27469, Korea; 3 Department of Aeronautical & Mechanical Design Engineering, Korea National University of Transportation, Chungju 27469, Korea; 4 Yonsei Cancer Center, Division of Medical Oncology, Department of Internal Medicine, Graduate School of Medical Science Brain Korea 21 Project, College of Medicine, Yonsei University, Seoul 03722, Korea; 5 College of Pharmacy, Chosun University, 309 Pilmun-daero, Dong-gu, Gwangju 61452, Korea

**Keywords:** cancer-derived exosome, magnetofluoro-immunoassay, cancer diagnosis, prostate cancer, binary nanoparticle-decorated graphene, magnetoplasmonic graphene

## Abstract

Multi-functionalized carbon nanomaterials have attracted interest owing to their excellent synergic properties, such as plasmon resonance energy transfer and surface-enhanced Raman scattering. Particularly, nanoparticle (NP)-decorated graphene (GRP) has been applied in various fields. In this study, silver NP (AgNP)- and magnetic iron oxide NP (IONP)-decorated GRP were prepared and utilized as biosensing platforms. In this case, AgNPs and GRP exhibit plasmonic properties, whereas IONPs exhibit magnetic properties; therefore, this hybrid nanomaterial could function as a magnetoplasmonic substrate for the magnetofluoro-immunosensing (MFI) system. Conversely, exosomes were recently considered high-potential biomarkers for the diagnosis of diseases. However, exosome diagnostic use requires complex isolation and purification methods. Nevertheless, we successfully detected a prostate-cancer-cell-derived exosome (PC-exosome) from non-purified exosomes in a culture media sample using Ag/IO-GRP and dye-tetraspanin antibodies (Ab). First, the anti-prostate-specific antigen was immobilized on the Ag/IO-GRP and it could isolate the PC-exosome from the sample via an external magnetic force. Dye-tetraspanin Ab was added to the sample to induce the sandwich structure. Based on the number of exosomes, the fluorescence intensity from the dye varied and the system exhibited highly sensitive and selective performance. Consequently, these hybrid materials exhibited excellent potential for biosensing platforms.

## 1. Introduction

In the past few decades, hybrid nanomaterials, such as core@shell nanomaterials and nanoparticle (NP)-modified carbon nanomaterials, have been highlighted owing to their synergistic and multifunctional properties, such as surface-enhanced Raman scattering (SERS), magneto-optical effects, plasmon resonance energy transfer, and enhanced localized surface plasmon resonance [1,2,3]. These nanomaterials were employed for the development of high-performance biosensing systems [4,5,6,7]. Among the various types of hybrid nanomaterials, magnetic and metal NP-based hybrid nanomaterials were employed for detecting viruses and deoxyribonucleic acid (DNA) through magnetofluoro-immunosensing (MFI) platforms [8,9]. Furthermore, NP-modified graphene possesses magnetic and plasmonic properties, which enable it to function as both a magnetoplasmonic (which can capture and isolate target biomolecules using an external magnetic force) and a plasmonic substrate [10].

Exosomes are considered potential diagnostic biomarkers because they can be obtained from diverse cell lines (e.g., cancer, stem, and immune cells) and various body fluids (e.g., urine, cerebrospinal fluid, and blood) [11,12,13]. They comprise tetraspanins, disease-specific antigens, and signaling receptors that are embedded in the lipid bilayer membrane. In addition, secreted exosomes may be able to trap disease-specific ribonucleic acid (RNA) so that they can be applied as biomarkers for biopsy-based diagnosis [14,15]. Interestingly, some exosomes exhibit different characteristics depending on their source. For example, the prostate-cancer-cell-derived exosome (PC-exosome) contains prostate-specific antigen (PSA), whereas ovarian-cancer-cell-derived exosome contains CD24 and EpCAM [16,17,18]. Due to the cancer marker including PSA that can be found on the surface of the PC-exosome, some research groups have tried to detect this exosome by using target antibodies, such as anti-PSA, through the sandwich ELISA approach [19]. Therefore, many scientists have tried to develop exosome-based cancer diagnostic systems.

These exosomes exhibit excellent potential for use as biomarkers; nevertheless, the isolation and purification of disease-specific exosomes is difficult and complex because the number of disease-specific exosomes is low [20,21]. In addition, the stability of exosomes is of high concern because it reduces after purification and isolation [22]. These issues could contribute to poor diagnostic performance.

Therefore, we aimed to directly detect cancer-cell-derived exosomes via the MFI system using Ag/iron oxide NP-decorated graphene (Ag/IO-GRP) without purification and concentration processes. The MFI-based sensing process is described below. First, the anti-PSAs were modified on the Ag/IO-GRP surface via the 1-ethyl-3-(3-dimethylaminopropyl)carbodiimide/N-hydroxy succinimide (EDC/NHS) reaction for use as magnetoplasmonic substrates. Conversely, PC-exosomes containing culture media were obtained after an LNCaP.FGC cell culture; moreover, the LNCaP.FGC-derived exosome (LF-exo), which possessed PSA, which functioned as a PC biomarker. Subsequently, anti-PSA-Ag/IO-GRP was added to the culture media sample containing LF-exo to capture the target exosome, and magnetic separation was conducted to isolate the LF-exo captured by the hybrid structure. Next, AF488-dye-labeled CD9 tetraspanin antibody (AF488-CD9 Ab) was added to the magnetically isolated sample to measure the fluorescence (FL) intensity as a function of the concentration of PC-exosomes. The sensitivity of the MFI system was performed with PC-exosome concentrations ranging from 10^2^ NPs/mL to 10^6^ NPs/mL, and the selectivity test was also conducted with normal cell-derived exosomes and cow exosomes in fetal bovine serum (FBS) and culture media mixture; based on the sensing results, the MFI system exhibited high sensitivity and selectivity performance.

## 2. Results

**Figure 1:**
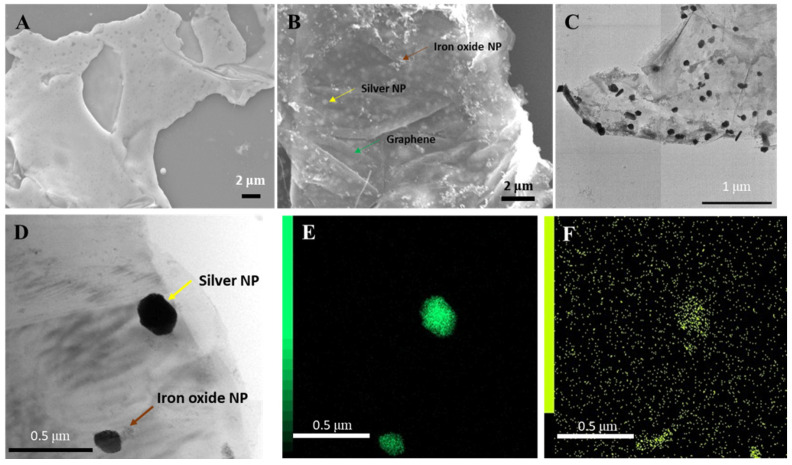
Structure observation of Ag/IO-GRP: SEM images of (**A**) bare GRP and (**B**) Ag/IONP-GRP, TEM image of (**C**) Ag/IO-GRP and EDS results, (**D**) bright field image, (**E**) Ag L mapping image, and (**F**) Fe K mapping image of Ag/MNP-GRP.

The structure of the Ag/IO-GRP hybrid nanomaterial was observed using scanning electron microscopy (SEM) and transmission electron microscopy (TEM). Prior to the NP decoration process, a clean and smooth GRP surface was shown using SEM (Figure 1A), and the spherical iron oxide NPs (IONPs) were observed using TEM (size: ~12 nm) (Figure S1). After the decoration process, two different types of NPs were observed on the graphene surface using SEM and TEM and were dispersed on the graphene surface (Figure 1B,C). Based on the TEM images, black particles and small gray particles denoting AgNPs and IONPs (Figure 1C,D), respectively, were observed. Since the electron density of an AgNP is higher than that of an IONP, the color of the AgNPs was darker than that of the IONPs (size: ~100 nm). In addition, the elemental analysis of the Ag/IO-GRP surface was performed using energy-dispersive X-ray spectroscopy (EDS) with TEM and SEM. Based on the EDS spectrum in Figure S2, the energy levels of Ag and Fe were clearly measured, indicating that AgNPs and IONPs were successfully dispersed on the GRP surface. In particular, according to the mapping results in Figure 1E,F, the Fe K density was accumulated near the Ag L energy density, and the antioxidant IONP could reduce the Ag ions to AgNPs and they were co-deposited on the GRP surface.

**Figure 2:**
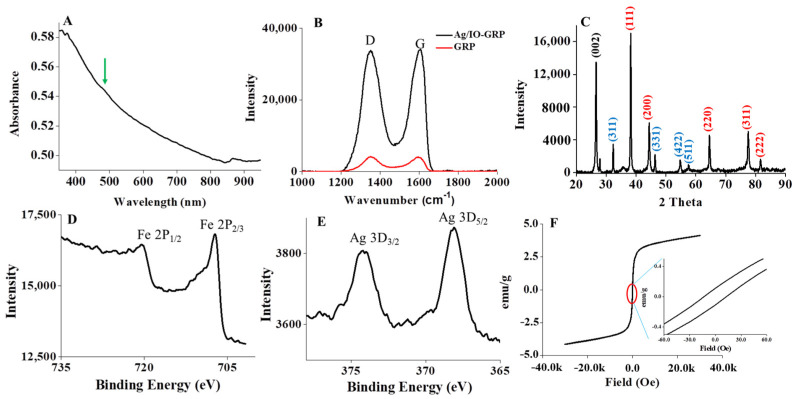
Physicochemical properties of Ag/IO-GRP: (**A**) UV/Vis spectrum, (**B**) Raman spectra, (**C**) XRD diffraction pattern, (**D**,**E**) XPS spectra, and (**F**) SQUID hysteresis.

The physicochemical properties of Ag/IO-GRP were characterized and are shown in Figure 2. First, the plasmonic absorbance of AgNPs on the hybrid structure was measured at ~480 nm using UV/Vis spectroscopy (green arrow in Figure 2A). This result indicated that large AgNPs were formed on the GRP surface and it was well matched with the TEM result. In addition, the SERS effect of Ag/IO-GRP was clearly measured using Raman spectroscopy at an excitation wavelength of 532 nm (Figure 2B). In both spectra, the D and G bands were measured at ~1352 cm^−1^ and 1603 cm^−1^, respectively. In this case, the Raman intensity of Ag/IO-GRP was much higher than that of GRP; therefore, the SERS effect was obviously characterized and it was demonstrated that this hybrid structure could function as a plasmonic substrate for the MFI system for biosensing applications. Following the amine functionalization of Ag/IO-GRP for antibody conjugation, the functional groups were characterized via Fourier transform infrared (FTIR) spectroscopy (Figure S3). The Fe-O vibrations and C-O bond were observed at ~594 cm^−1^ and ~1020 cm^−1^, respectively. In addition, C-N vibrations were observed at ~1213 cm^−1^. Conversely, the aromatic group of Ag/IO-GRP was identified at ~1378–1572 cm^−1^. The diffraction pattern of graphene, Ag, and IONPs in the hybrid structure was analyzed using powder X-ray diffractometer (pXRD, Figure 2C). First, the (002) diffraction peak at 2θ = 26.2° was shown due to the graphene structure (ICDD card no: 01-075-1621). In addition, XRD patterns of the IONPs from the (311), (331), (422), and (511) planes were analyzed at 2θ = 32.3°, 45.93°, 54.75°, and 57.54°, respectively (JCPDS card no: 79-0417). Furthermore, several diffraction patterns of AgNPs, including (111), (200), (220), (311), and (222), were observed at 2θ = 38.14°, 44.15°, 64.59°, 77.49°, and 81.66°, respectively (ICSD card no: 00−004−0783). The element analysis of Ag/IO-GRP was performed using X-ray photoelectron spectroscopy (XPS); the results are shown in Figure 2D,E. The existence of IONP was confirmed via the double peaks of Fe 2P_1/2_ and Fe 2P_2/3_, which were observed at approximately 720 eV and 707 eV, respectively. In addition, two Ag d orbital-related peaks for 3D_3/2_ and 3D_5/2_ were measured at approximately 374 eV and 368 eV, respectively; these peaks confirmed that the AgNPs were successfully dispersed on the GRP surface. The magnetic property of Ag/IO-GRP was characterized using superconducting quantum interference device (SQUID) analysis and the magnetic hysteresis curve of Ag/IO-GRP was clearly observed. The remanence effect was observed at approximately ±0.1 emu/g and the coercive force was approximately ±13 Oe.

In general, the number of exosomes was counted using NP tracking analysis (NTA), which is considered a normal method. However, using this technique, other proteins with similar densities and sizes to the exosomes were also counted, thus making it difficult to obtain the number of exosomes alone. In addition, it was hard to count the number of exosomes in the non-purified sample via NTA; therefore, we used another technique to obtain the number of exosomes.

**Figure 3:**
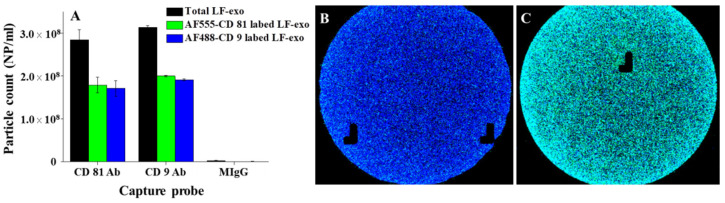
Tetraspanin characterization of PC-exosomes: (**A**) concentration of LF-exos and FL images of a dye-tetraspanin-labeled exosome (**B**) on a CD81 capture spot and (**C**) on a CD9 capture spot.

In this study, the number and tetraspanin markers of LF-exo were characterized via ExoView analysis. In this case, first, the exosome sample was dropped onto the sensing chip containing tetraspanin Abs and MIgG (negative control) immobilized capture spots, and then the exosomes captured on each spot were labeled with dye-conjugated tetraspanin Ab for the particle counts and colocalization analysis. According to the results in Figure 3A, the total number of LF-exos counted was approximately 2.8 × 10^8^ NPs/mL and 3.1 × 10^8^ NPs/mL at the CD81 and CD9 capture spots, respectively. In addition, it was confirmed that CD81 and CD9 coexisted on the single exosome via the colocalization count based on the numbers of AF488-conjugated CD9 Ab (AF488-CD9)- and AF555-conjugated CD81 Ab (AF555-CD81)-labeled exosomes at the CD81 and CD9 Ab spots, respectively (Figure 3B,C). Furthermore, the FL images shown in Figure 3B,C revealed that the numerous cyan color spots indicated AF488-CD9 (blue)- and AF55-CD81 (green)-labeled exosomes, respectively. LF-exos were also characterized using Western blotting (Figure S4). Based on the results, the CD9 and CD81 bands acting as tetraspanin markers and Alix as cargo protein in the exosomes were clearly measured; therefore, both tetraspanins could participate in sensing applications as FL signal transducers.

**Figure 4:**
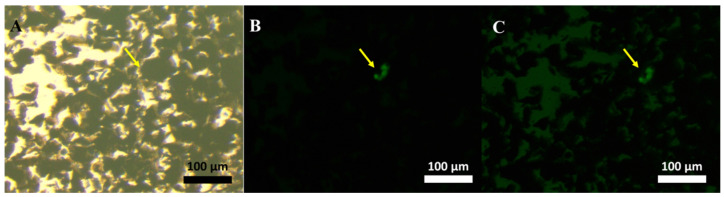
FL images after LF-exo conjugation with Ag/IO-GRP and AF488-CD9 via antibody and exosome binding: (**A**) bright field image, (**B**) FL image, and (**C**) bright field and FL merged condition image.

The binding between PSA Ab and Ag/IO-GRP was checked using an ELISA test and it was confirmed that Ab was successfully modified on the surface of Ag/IO-GRP (Figure S5). To demonstrate the exosome-sensing performance, first, an assembly structure of LF-exo-mediated Ag/IO-GRP and AF488-CD9 was magnetically isolated using an external magnetic force and its structure was observed using FL microscopy (Figure 4). In this case, the green FL was clearly exhibited on the graphene structure (yellow arrow in Figure 4B,C) and it was demonstrated that this MFI-based cancer-cell-derived exosome detection was successfully conducted. In addition, this revealed the potential for the visual detection of exosomes.

**Figure 5:**
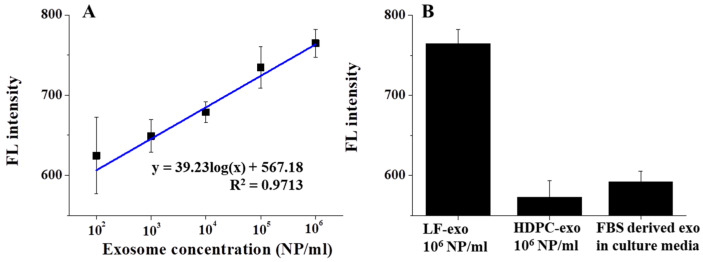
Disease-specific exosome detection performance based on a magnetofluoro-immunoassay using Ag/IO-GRP and AF488-CD9: (**A**) sensitivity test and (**B**) selectivity test against other exosomes.

The sensitivity test was performed by measuring the FL intensity as a function of the number of exosomes ranging from 10^2^ NPs/mL to 10^6^ NPs/mL after magnetic isolation using AF488-CD9. In general, the amount of exosome-related protein-based sensing performances was conducted for sensitivity tests; however, in our study, the FL difference was monitored with different concentrations of the exosome itself. According to Figure 5A, the FL intensity was correlated with the number of LF-exos and the exosome concentration increased as the FL intensity gradually increased, thus yielding a linear response from the MFI process. In this case, the LOD was calculated to be around 134.32 NPs/mL using Equation (S1) in the Supplementary Information. 

Conversely, the sensitivity test of the MFI system was also performed using AF555-CD81 with the same concentration range of LF-exos (Figure S6). In this case, the FL intensity also gradually changed depending on the amount of LF-exos. However, the R^2^ value of this case was 0.8934, which was considerably lower than that of the AF488-CD9 case; therefore, a lower sensing performance was observed. As previously mentioned, this result indirectly indicated that CD9 tetraspanin was more highly expressed than other tetraspanins in cancer-derived exosomes.

The selectivity test was performed with other exosomes, such as human-dermal-papilla-cell-derived exosome (HDPC-exo) and cow exosomes in a mixture of FBS and culture media (Figure 5B). In this case, the same amount (10^6^ NPs/mL) of HDPC-exos and LF-exos was introduced to the MFI system to evaluate the selectivity; however, only the LF-exos exhibited a high response. Conversely, it is well known that high amounts of FBS-derived exosomes exist in cell culture media and function as critical impurities for exosome-based nanobio applications [23]. Therefore, considerable funds were expended to obtain FBS-derived exosome-free agents and FBS-derived exosome depletion methods were utilized for bioapplications [24,25]. However, in our study, FBS-derived exosomes in culture media mixtures could not hinder the detection of LF-exos and it was demonstrated that there was no change in FL under the FBS-culture media condition. Therefore, this Ag/IO-GRP-based MFI system for detecting cancer-derived exosomes exhibited excellent sensing performance and a high potential for use in biosensing systems.

In addition, the sensing performance of Ag/IO-GRP was compared with that of the GRP structure, and the sensitivity result using GRP is shown in Figure S7. According to this result, the FL intensity was increased at a high concentration of exosomes, but its sensing performance was not good and it was not a linear response. Of course, the GRP also has a plasmonic property; therefore, it could be utilized as a plasmonic substrate for a fluoro-immuno sensing system. However, since it does not possess a magnetic property, the captured LF-exo could not be isolated from the impurities using an external magnetic force. Therefore, using GRP alone is not suitable for a magnetofluoro-immunosensing platform to detect the non-purified sample.

Recently, various approaches for exosome detection were investigated and developed with nanomaterial-assisted sensing systems, and our detection method was compared with other studies (Table 1). In some other studies, exosomes were detected in pure environments without impurities. However, in this study, non-purified exosomes were successfully monitored, and interestingly, the LOD of this system was improved compared with that of other detection platforms. This indicated that this system could be considered a potential diagnosis method for cancer-derived exosomes.

## 3. Discussion

The Ag/IO-GRP was prepared using a facile two-step synthesis method (Figure S8). First, GRP was mixed with Ag^+^ ions in the DI water via sonication, then an antioxidant gallic-acid-modified IONP (GA-IONP) solution was added to the mixture and stirred for 3 h. In particular, in the second step, the GA-IONP functioned as a reducing agent to synthesize AgNPs from the Ag^+^ ion on the GRP surface. Therefore, the NP decoration process on the GRP was possible without any reducing chemicals, such as NaBH_4_ or hydrazine. In addition, the GA-IONPs also could be attached to the surface of the GRP through the π–π interactions due to the aromatic group of GA on the IONP surface. Finally, the Ag/IO-GRP was synthesized using this strategy. Furthermore, because the AgNPs and GRP exhibit plasmonic properties and IONPs exhibit magnetic properties, this hybrid structure could be applied as the magnetoplasmonic substrate and this synergic property could be used for a magnetofluoro-immunoassay.

As mentioned above, exosomes are considered as cancer biomarkers and are used for diagnosis; however, in many cases, exosomal RNA and cargo proteins, rather than the exosome itself, participate as target biomolecules. To diagnose cancer, the extraction of target biomolecules from isolated exosomes was performed, which required additional time and cost. Moreover, it was an extremely difficult process.

**Figure 6:**
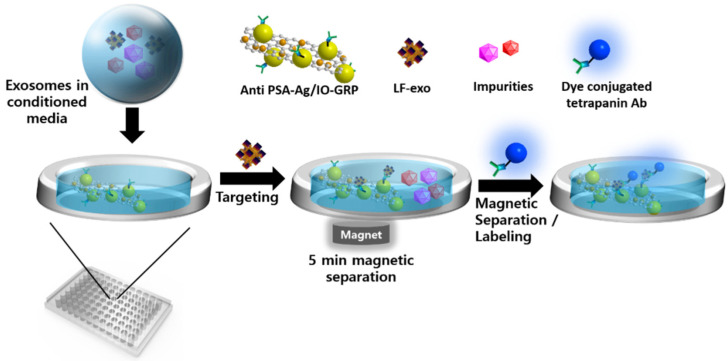
Schematic illustration of a Ag/IO-GRP-based MFI system for the detection of cancer-cell-derived exosomes.

To overcome these hurdles, in this study, we directly detected cancer exosomes using a sample that was not purified. A more facile detection process was suggested as part of our study. The exosome detection process of a Ag/IO-GRP-based MFI system is illustrated in Figure 6. In this case, PSA-containing LF-exos in the non-purified culture media was added to the Ag/IO-GRP matrix and mixed via shaking. During this step, LF-exos were bound with anti-PSA-conjugated Ag/IO-GRP, the exosome-hybrid nanomaterial complex was magnetically isolated, and the non-binding impurities were removed via PBS washing. Second, AF488-CD9 or AF555-CD81 was added to the complex solution to induce the dye-conjugated tetraspanin-exosome-Ag/IO-GRP sandwich structure. FL was measured based on the LF-exo concentration. Actually, it was found that CD9 on an exosome could promote PC cell growth, indicating that CD9 could be a key exosome biomarker [29,30]. Therefore, in our study, AF488-CD9 functioned as a signal inducer to detect LF-exos. Furthermore, according to the sensitivity result based on CD9-positive LF-exo, a linear response was clearly obtained with a low LOD. Conversely, CD81 is also one of the tetraspanins on the exosome; therefore, AF555-CD81 was also used as an FL-monitoring agent for the MFI system to detect the Ag/IO-GRP-captured LF-exos. In this case, the linear sensitivity was measured but its accuracy was not as high as in the CD9 case. It was confirmed that CD9 is a key exosomal biomarker.

The stability of the sensing material is also an important factor, and in our previous studies, gold/IO-GRP, which has a similar structure to Ag/IO-GRP, played a role of a plasmonic and electrical substrate for the detection of viruses, and that structure showed excellent stability as a sensing platform [31,32]. Thus, these kinds of magnetoplasmonic graphene materials could be used for a universal sensing platform to detect various viruses, exosomes, and so on.

## 4. Materials and Methods

### 4.1. Materials and Instruments

AgNO_3_, gallic acid (GA; 3,4,5-trihydroxy benzoic acid), FeCl_3_, FeCl_2_·4H_2_O, and 25% NH_4_OH solution were purchased from Sigma-Aldrich. Graphene was obtained as standard graphene (Ulsan, South Korea) and graphene oxide (GRP) was prepared via Hummers’ method using H_2_SO_4_ and HNO_3_. The prostate cancer cells, namely, LNCaP.FGCs, were purchased from the Korean Cell Line Bank (Seoul, South Korea) and the RPMI 1640 culture medium was purchased from Thermo Fisher Scientific (Seoul, Korea). Anti-PSA (PSA Ab) was purchased from AB Chem. ExoView Tetraspanin Kits containing tetraspanin antibody dyes (AF488-anti CD9 and AF555-anti CD81) were obtained from NanoView Biosciences (Boston, MI, USA). The absorbance of Ag/IO-GRP was measured using UV/Vis spectroscopy (Lambda 750, Perkin Elmer, Hopkinton, MA, USA). The functional groups of the hybrid structure were monitored using FTIR spectroscopy (Cary 610, Agilent Technologies, Santa Clara, CA, USA). A pXRD (RINT ULTIMA, Rigaku Corp., Tokyo, Japan) was used to characterize Ag/IO-GRP with Cu Kα radiation and a Ni filter. The patterns were collected from 2θ = 20–90° at a scan rate of 0.01° per step and 10 s per point. The element analysis of Ag/IO-GRP was conducted using XPS (ESCA3100, Shimadzu, Kyoto, Japan). The magnetic property of the particles was analyzed using the Magnetic Property Measurement System (MPMS3-Evercool, Quantum Design, San Diego, CA, USA). The morphologies and sizes of the NPs and hybrid carbon nanomaterials were observed using high-resolution transmission electron microscopy (HR-TEM, JEM-2100F, JEOL, Tokyo, Japan) and scanning transmission electron microscopy (STEM, EVO MA 10, Carl Zeiss, Oberkochen, Germany). The FL image of the AF488-CD9/PC-exosome/Ag/IO-GRP structure was observed via FL microscopy with 200× magnification (IX73, Olympus, Tokyo, Japan). The FL of the dye in the magnetofluoro-immunoassay system was measured using a microplate reader (Synergy HTX, Agilent Technologies, Santa Clara, CA, USA).

### 4.2. Synthesis of GA-IONPs and Ag/IO-GRP

GA-IONPs were prepared via a coprecipitation process. FeCl_3_ (1 mmol, 0.1622 g) and FeCl_2_·4H_2_O (0.5 mmol, 0.0994 g) were dissolved in deionized (DI) water (20 mL). Then, 25% NH_4_OH solution (0.6 mL) was added to the mixture and stirred for 10 min to form IONPs. Subsequently, GA (1.5 mmol, 0.255 g) powder was poured into the black IONP solution and stirred at 90 °C for 30 min. Owing to the binding between GA and Fe in the GA-IONP structure, the solution color changed from black to deep violet. After stirring, the GA-IONPs were purified and precipitated with excess acetone and separated using a magnet.

In addition, Ag/IO-GRP was synthesized via two simple steps [31]. First, 0.2 mmol of AgNO_3_ was dispersed with 2 mg of GRP in 40 mL of DI water for 30 min under sonication. Subsequently, 1 mL of GA-IONP solution (2 mg/mL concentration in DI water) was dropped into the mixture. Ag/IO-GRP was obtained after vigorous stirring for 3 h. The hybrid nanomaterials were separated via a magnetic force and dried in the oven at 50 °C.

### 4.3. Preparation of Exosomes from the Prostate Cancer Cell

To detect disease-specific exosomal biomarkers through a magnetofluoro-immunoassay, non-purified LF-exos were prepared. First, LNCaP.FGC cells were seeded and cultured with Roswell Park Memorial Institute (RPMI) 1940 media + 10% FBS + 1% PNC + 1% NEAA + 0.1% 2-mercaptoethanol in the 100 mm cell culture dish. After 48 h of cell cultivation, the media was collected and centrifuged at 1500 rpm for 10 min to remove the cell debris and large impurities. The supernatant was used as the non-purified LF-exo sample and was characterized using the ExoView platform to confirm the particle counts and tetraspanin content of the LF-exos. In addition, its tetraspanins were characterized through Western blotting; the detailed methods are described in the Supporting Materials.

### 4.4. PSA Antibody Modification of Ag/IO-GRP and Magnetofluoro-Immunoassay Performance for LF-Exo Detection

To modify the surface of Ag/IO-GPR with PSA antibodies, the Ag/IO-GRP surface was first modified by cysteamine for amine functionalization. In this case, 2 mg of Ag/IO-GRP was dispersed in 2 mL of DI water and mixed with 1 mL of 0.01 M cysteamine in 10 mL of DI water for 1 h and after functionalization, the hybrid structure was isolated and purified using an external magnetic force and dried in an oven at 50 °C.

The PSA Ab was conjugated with amine-functionalized Ag/IO-GRP via an EDC/NHS coupling reaction in a 96-well plate. First, amine-functionalized Ag/IO-GRP (2 mg/mL, 30 µL) was mixed with EDC (50 mM, 30 µL) and NHS (50 mM, 30 µL), and the mixture was shaken for 1 min under 200 rpm to prevent the unexpected coupling reaction between the carboxyl group and amine group of PSA Ab [32]. Subsequently, PSA Ab (30 µL) was added to the mixture and shaken for 3 h at 200 rpm. After the antibody conjugation, the hybrid structure was isolated using an external magnetic force and washed with phosphate-buffered saline (PBS). The conjugation between PSA Ab and Ag/IO-GRP was checked via an ELISA test.

The sensitivity and selectivity tests of magnetofluoro-immunoassay for LF-exo detection were performed by measuring the FL intensity as a function of the exosome concentration. To conduct the sensitivity test, 100 μL of non-purified LF-exos from 10^2^ NPs/mL to 10^6^ NPs/mL were added to PSA-Ab-conjugated Ag/IO-GRP solution and the mixture was incubated for 1 h under 90 rpm shaking. The LF-exo-captured Ag/IO-GRPs were isolated using an external magnetic force for 5 min and washed with PBS; then 100 μL of AF488-conjugated CD9 antibody (AF488-CD9) and AF555-conjugated CD81 antibody (AF555-CD81) were added to the mixture and shaken for 1 h at 90 rpm. Again, the labeled LF-exo-captured Ag/IO-GRPs were separated using a magnet and washed with PBS. Lastly, the FL intensity was measured with an excitation wavelength at 485 nm and the intensity was collected at 528 nm for AF488-CD9. In addition, AF555-CD81-labeled LF-exo-Ag/IO-GRP was excited at 530 nm to measure the FL intensity and its emission was analyzed at 590 nm. The limit of detection (LOD) was confirmed using Equation (S1) in the Supplementary Materials. In contrast, the selectivity test was performed using 10^6^ NPs/mL of HDPC-exo and FBS mixed culture media containing the FBS-derived exosome.

## 5. Conclusions

The Ag/IO-GRP was synthesized in DI water without harsh conditions, such as high temperature and toxic reagents, and it successfully functioned as a magnetoplasmonic substrate for detecting cancer-cell-derived exosomes using the MFI process with the assistance of the tetraspanin dye structure. In particular, non-purified LF-exos were detected via the MFI system, and a linear sensitivity was observed as a function of the concentration of LF-exos (10^2^–10^6^ NPs/mL). The LOD of this system was estimated to be around 134.32 NPs/mL; therefore, excellent sensitivity was exhibited. In addition, its selectivity against several types of exosomes was demonstrated. Therefore, the Ag/IO-GRP-based MFI platform demonstrated excellent potential as a biosensor and diagnostic system for complex biological samples. Based on our study results, diagnosis via MFI using disease-specific exosomes from clinical samples will be demonstrated in further research.

## Figures and Tables

**Table 1:** Comparison of recent research into exosome detection via various detection methods.

Detection Method	Target Exosome	Detection Range/LOD	Ref.
SERS-based detection	LNCaP-derived exosome	1.8 × 10^2^–1.8 × 10^7^ NPs/μL	[26]
		203 NPs/μL	
Aptamer assisted colorimetry	MCF-7-derived exosome	5.6 × 10^4^–8.9 × 10^5^ NPs/μL	[27]
		4.5 × 10^3^ NPs/μL	
Localized surface plasmonic resonance-based detection	PD-L1-positive exosome	1.2 × 10^3^–6.2 × 10^3^ NPs/μL	[28]
		1.2 × 10^3^ NPs/μL	
FRET-based magnetofluoro-immunoassay	Exosome in serum	0.88–8.80 mg/mL (total protein of exosome)	[11]
		0.28 mg/mL	
Magnetofluoro-immunoassay	Non-purified LF-exo	10^2^–10^6^ NPs/mL	This work
		134.32 NPs/mL	

## Supplementary Materials

The supporting information can be downloaded from https://www.mdpi.com/article/10.3390/ijms23179619/s1. References [33,34] are cited in the Supplementary Materials.
