# Supplementary material for: Magnetofluoro-Immunosensing Platform Based on Binary Nanoparticle-Decorated Graphene for Detection of Cancer Cell-Derived Exosomes
Source: Int J Mol Sci. 2022 Aug 25;23(17):9619. doi: 10.3390/ijms23179619 (PMC9455968; doi:10.3390/ijms23179619)
Supplement: Supplementary file 1 [file ijms-23-09619-s001.zip › ijms-1883191-supplementary.pdf]

Supplementary Information

Magnetofluoro-immunosensing platform based on binary nanoparticle-decorated graphene for detection of cancer cell-derived exosomes

Jaewook Lee[1,*,†], Ji-Heon Lee[1], Jagannath Mondal[2], Joon Hwang[1,3], Han Sang Kim[4], Vinoth Kumar[1,‡], Akhil Raj[5], Seungrim Hwang[5], and Yong-Kyu Lee[1,2**]

[1]4D Convergence Technology Institute (National Key Technology Institute in University), Korea National University of Transportation, Jungpyeong, Chung-Buk, 27909, Republic of Korea.

[2]Department of Chemical and Biological Engineering, and [3]Department of Aeronautical & Mechanical Design Engineering, Korea National University of Transportation, Chungju, Chung-Buk 27469, Republic of Korea.

[4]Yonsei Cancer Center, Division of Medical Oncology, Department of Internal Medicine, Graduate School of Medical Science Brain Korea 21 Project, Yonsei University College of Medicine, Seoul, Republic of Korea

[5]College of Pharmacy, Chosun University, 309 Pilmun-daero, Dong-gu, Gwangju 61452, Republic of Korea.

[†]Current affiliation: Lee Gil Ya Cancer and Diabetes Institute, Gachon University, Incheon, 21999, Republic of Korea

[‡]Current affiliation: Magee-Womens Research Institute, Department of Obstetrics, Gynecology and Reproductive Sciences, University of Pittsburgh, Pittsburgh, PA 15213, USA

Correspondence: nanojaewook@gmail.com (J.L.) and leeyk@ut.ac.kr (Y.-K.L.)

[Supplementary method]

1. Purification of LF-exosome through ultracentrifugation for western blot test.

Conditioned media was collected from the 80% confluent LNCaP.FGC prostate cancer cell culture. Then the media has been centrifuged in low-speed relative centrifugal forces (RCF)

such as 300 RCF and 2500 RCF each for 10 minutes then 16500 RCF for 20 minutes to remove the pelleted dead cells and cell debris by using the VS-550, Multitube carrier refrigerated centrifuge from Vision Scientific Co. Ltd, Republic of Korea South. After the filtration of this supernatant through a bottle top filter (500 mL rapid flow bottle top filter, 0.2 µm aPES membrane, 75 dia. 45 mm neck from Nalgene rapid flow filters, Thermo Scientific, Waltham, Massachusetts, United States) to eliminate the nanoparticles which come in the size more than 200 nm, ultra-centrifuged (UC) the filtrate in 100,000 RCF for 2 hours at 4˚C by using VS 30,000i Superspeed vacuum centrifuge from Vision Scientific Co. Ltd, Republic of Korea South, to pellet the exosome. The resulting exosome pellet has been purified with 1X Phosphate Buffered Saline (PBS, 0.067 M, Hyclone™, Cytiva from the United States) by one more UC in 100,000 RCF for 2 hours at 4 ˚C. Finally, the exosome pellet was reconstituted in 200 µL of 1X PBS and kept overnight at 4 ˚C with slight shaking. This LF-exosome sample was stored at -80 ˚C for further analysis.

2. Characterization of LF-exosome by western blot

LNCaP.FGC prostate cancer cell lysates were prepared from 100 mm cell culture dish with 80% of growth confluence. The protein quantification of both isolated exosome, and the cell lysates were estimated using bicinchoninic acid (BCA) assay. Here exactly 15 µg of LF-exosome and 15 µg of cell lysates were loaded to 10 % polyacrylamide gel along with the molecular protein ladder. The gel ran for the first 1 hour at 60 V and the rest at 80 V. After the clear separation of the molecular ladder, protein transfer has been done to a polyvinylidene difluoride (PVDF) membrane in normal horse serum for 1 hour 30 minutes at 100 V. After the protein transfer, the PVDF membrane has been subjected to immunoblotting with 1: 1000 (primary antibody: normal horse serum) overnight at 4 ˚C with 50 RPM shaking. Then after the 3 times washing with 1X Tris-*buffered* saline-0.1 % Tween® 20

Detergent (*TBST*) for 10 minutes at 110 RPM, the PVDF membrane was incubated with 1: 8000 (horseradish peroxidase (HRP) conjugated secondary antibody: skimmed milk solution) for 1 hour 30 minutes at 95RPM shaking. Again after 3 times washing with TBST buffer for 10 minutes at 120RPM, detection has been done with 1;1 chemiluminescent ECL substrate mix A and B by the instrument LAS imager 680, GE health care from Chicago, United States.

3. *Calculation of limit of detection (LOD)*

The LOD was estimated from the assessment of standard deviation of the blank and the LOD was calculated by the below equation. [1,2]

$$LOD = e^{((3 \times \delta blank)/g)} \qquad \text{(Equation S1)}$$

where δblank is the standard deviation of blank sample and g is slope of calibration curve.

[Supplementary images]

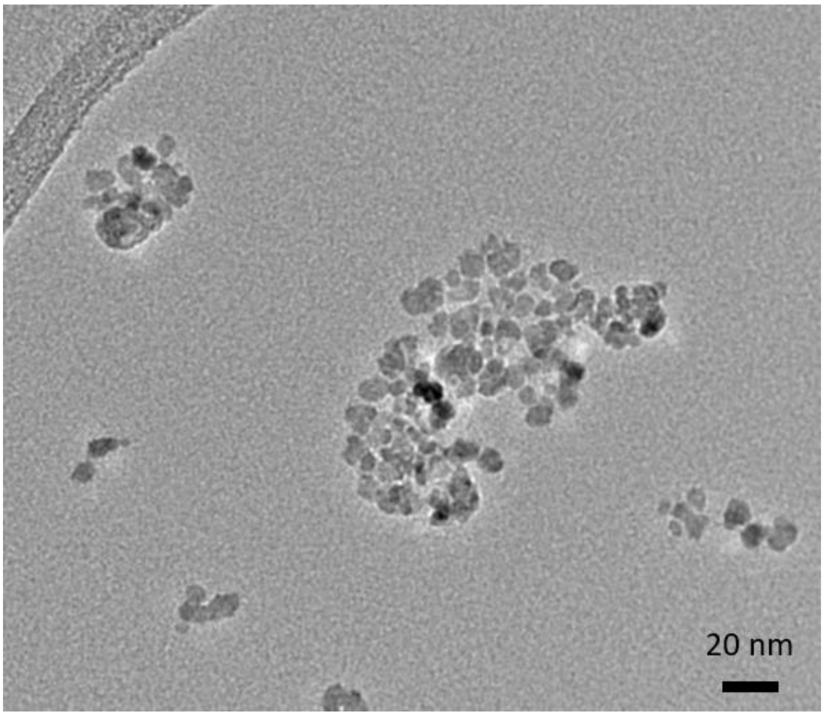

Figure S1. TEM image of GA-IONP

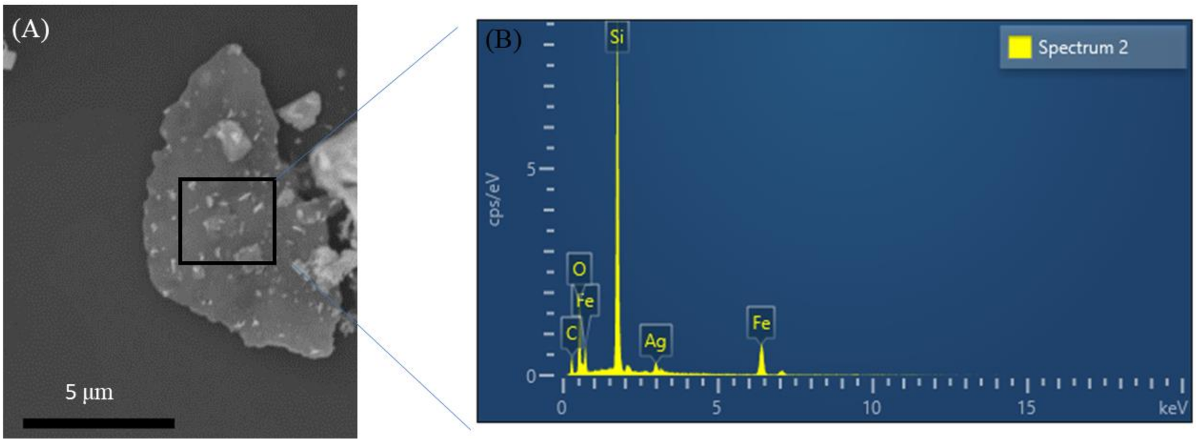

Figure S2. SEM image and EDS result of Ag/IO-GRP

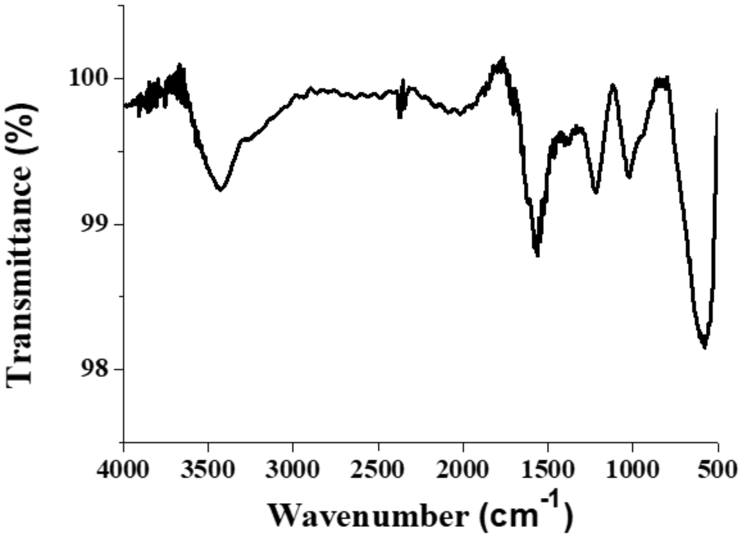

Figure S3. FT-IR spectroscopy of amine functionalized Ag/IO-GRP

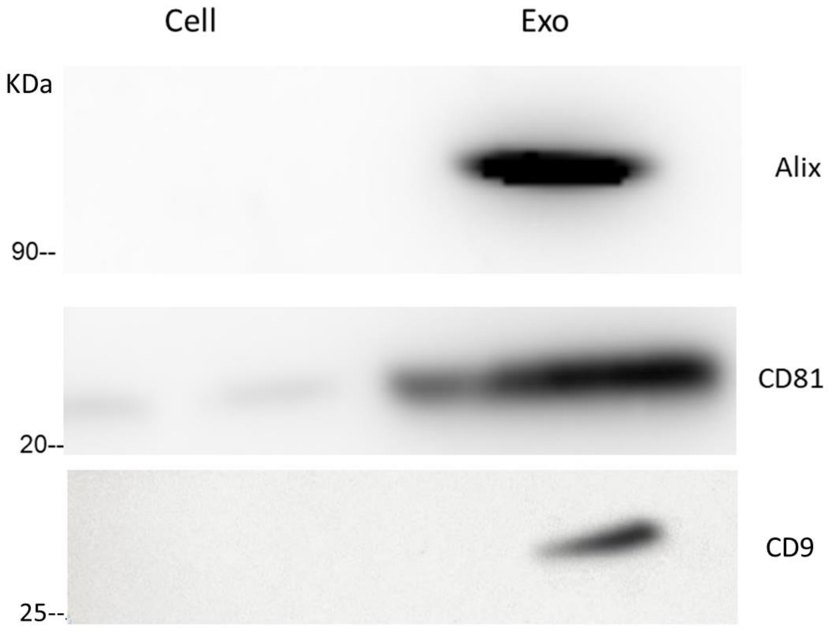

Figure S4. Analysis results of LF-exo marker by western blot

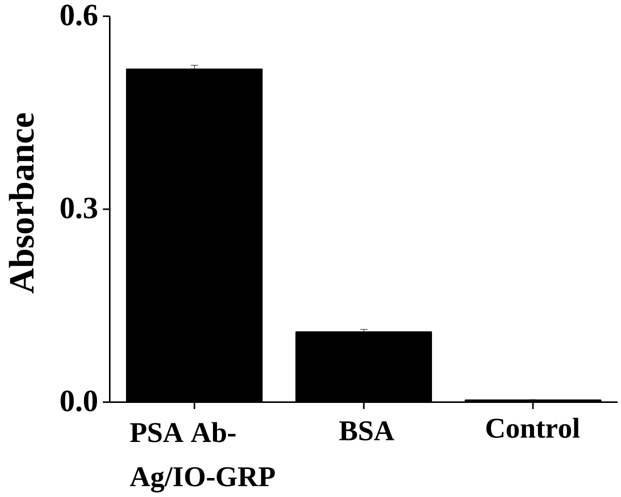

Figure S5. PSA Ab conjugation confirmation by ELISA test

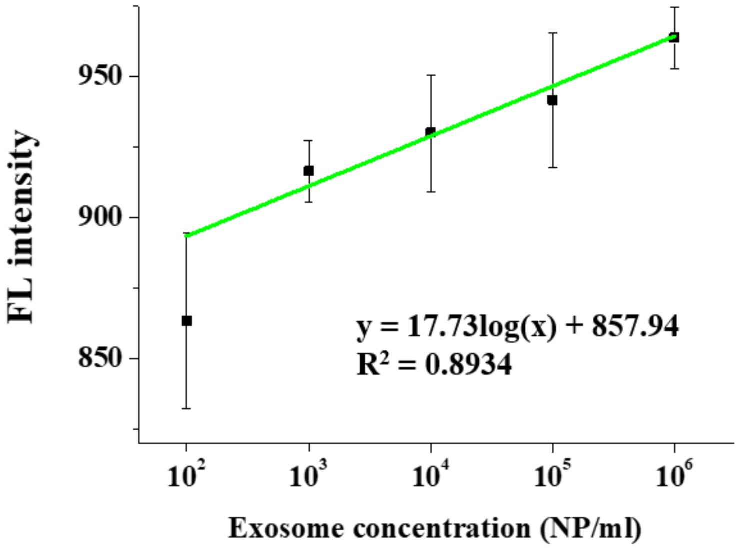

Figure S6. Sensitivity test using Ag/IO-GRP and AF 555-CD81.

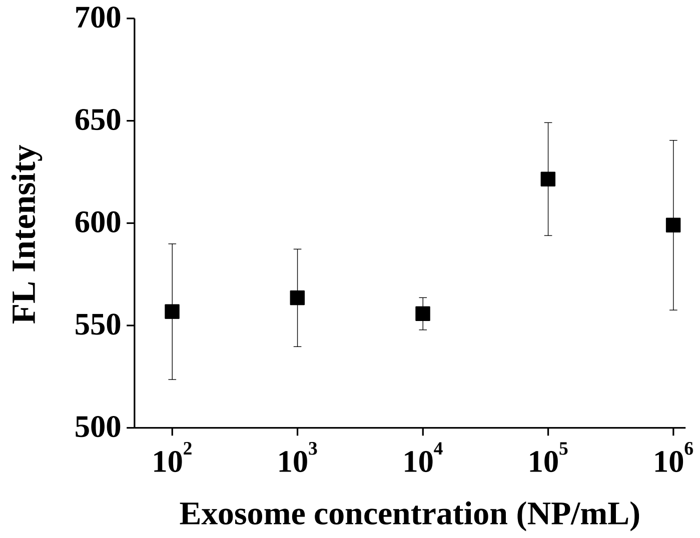

Figure S7. Sensitivity test with GRP and AF 488-CD9.

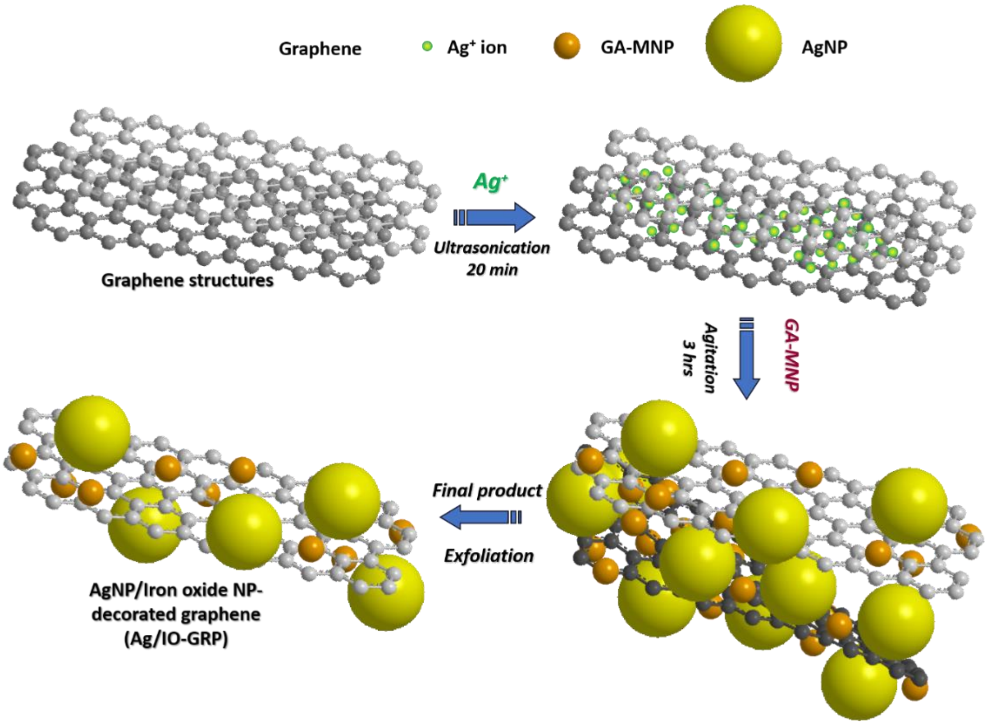

Figure. S8. Schematic illustration of Ag/IO-GRP synthesis

Supplementary Information Reference

1. Apostol, I.; Miller, K. J.; Ratto, J.; Kelner, D. N. Comparison of Different Approaches for Evaluation of the Detection and Quantitation Limits of a Purity Method: A Case Study Using a Capillary Isoelectrofocusing Method for a Monoclonal Antibody. *Anal. Biochem.* **2009,** *385*, 101-106

2. Lee, J.; Kim, J.;, Ahmed, S. R.; Zhou, H.; Kim, J.-M.; Lee, J. Plasmon-induced photoluminescence immunoassay for tuberculosis monitoring using gold-nanoparticle-decorated graphene, *ACS App. Mater. & Inter.* **2014**, *6*, 21380-21388
